# Transplant Center Attitudes Toward Early Liver Transplant for Alcohol-associated Liver Disease

**DOI:** 10.1097/TXD.0000000000001532

**Published:** 2023-08-28

**Authors:** Jonathan Mitchell, Kayleigh Herrick-Reynolds, Jennifer D. Motter, Mayan Teles, Olivia Kates, Hannah Sung, Po-Hung Chen, Elizabeth King, Andrew Cameron

**Affiliations:** 1 Department of Surgery, Johns Hopkins University School of Medicine, Baltimore, MD.; 2 Department of Surgery, Walter Reed National Military Medical Center, Bethesda, MD.; 3 Department of Medicine, Johns Hopkins University School of Medicine, Baltimore, MD.

## Abstract

**Background.:**

Many centers have removed 6-mo pretransplant alcohol abstinence requirements to provide early liver transplant (ELT) for individuals with severe alcohol-associated liver disease (ALD), but the practice remains controversial. Using data collected from a nationally distributed survey, this study examines the practices and attitudes of transplant centers in the United States regarding ELT.

**Methods.:**

A 20-item survey designed to assess center practices and provider attitudes was distributed to 225 medical and surgical directors from 143 liver transplant centers via email.

**Results.:**

Surveys were completed by 28.9% (n = 65) of directors and 39% (n = 56) of transplant centers. All responding centers reported evaluating patients for ELT. Circumstances for considering ELT included <6 mo of survival without a transplant (96.4%) and inability to participate in alcohol addiction therapy pretransplant (75%). Most (66%) directors indicated their center had established criteria for listing candidates with severe ALD for ELT. Regarding important factors for ELT candidate listing, 57.1% indicated patient survival, 37.5% indicated graft survival, and 55.4% indicated having a low risk of relapse. Only 12.7% of directors affirmed the statement, “Six months of pretransplant abstinence decreases the risk of relapse.”

**Conclusions.:**

More centers are providing ELT for severe ALD. Inability to participate in alcohol addiction therapy and <6 mo of survival are commonly reported circumstances for considering ELT. Continued investigation of posttransplant outcomes in patients receiving ELT is essential to establishing a national consensus for distributing this valuable resource.

Alcohol-associated liver disease (ALD) is a leading cause of liver failure requiring a liver transplant (LT). A considerable number of patients with severe ALD or alcohol-induced acute-on-chronic liver failure do not improve with medical therapy, and LT is the only therapeutic alternative.^1–4^ Pretransplant abstinence requirements have prohibited access to LT for this patient population, despite the high mortality associated with these diseases. This restriction to access stems from concerns surrounding the potential for alcohol relapse, reduced graft survival, and the fairness of allograft allocation to severe ALD patients.^1,5,6^ Recently, many centers have removed the pretransplant 6-mo abstinence requirement, providing early LT (ELT) for patients with severe ALD; however, the practice remains controversial.^7–9^ Despite this controversy, a recent survey of the public has shown that a majority of respondents were at least neutral toward ELT for patients with severe ALD,^10^ indicative of the continued evolution of opinions surrounding this practice.

The guidelines and practices surrounding listing and transplantation for ALD are institution specific. A 2015 survey by Hasanin et al found that only 27% of centers provided transplants for patients with severe ALD; this number increased to 51% in a 2018 study by Bangaru et al and further to 85% in a 2021 survey by Lim et al.^11–13^ Throughout the COVID-19 pandemic, there has also been an increase in reported episodes of harmful drinking, alcohol-related hospital admissions, and listing for ALD, with severe ALD accounting for a large number of these waitlist additions.^14–17^

As the pandemic-fueled rise in ALD-related LT continues and ELT for severe ALD has become more widely adopted across the United States,^14–17^ continued examination of the practices of transplant centers and attitudes of transplant providers is a key step in shaping a national consensus that determines the management of patients receiving this therapy. Using data collected from a nationally distributed survey, this study assesses the practices and attitudes of transplant centers in the United States regarding ELT for ALD.

## MATERIALS AND METHODS

### Survey Design

A 20-item survey was developed to assess individual center practices and attitudes toward ELT (Supplement 1, SDC, http://links.lww.com/TXD/A563). ELT was defined as an LT performed without 6 mo of alcohol abstinence. Elements of previously published surveys were incorporated for comparison and consistency.^11,12^ Center attitudes were assessed using questions answered on a 3-point Likert scale (Agree, Neutral, Disagree). To contrast the approach to ALD with the approach to other behavior-related causes of liver disease, the survey also evaluated center practices surrounding LT for nonalcoholic steatohepatitis (NASH). The survey was created in Qualtrics (Qualtrics, Provo, UT). This study was exempted by the Johns Hopkins Hospital Institutional Review Board (IRB00282599).

### Survey Administration

A list of LT centers from all 11 Organ Procurement and Transplantation Network (OPTN) regions was compiled in March 2020 from the OPTN member directory, with the exclusion of the 26 pediatric transplant centers.^18^ The survey was distributed to medical and surgical program directors of liver transplantation for each center who were identified using this directory. The survey was distributed via email between June 3, 2021, and September 12, 2021. For directors at each center who did not complete the survey after initial distribution, periodic email reminders were sent for up to 12 wk.

### Statistical Analysis

The data collected were analyzed at both the respondent level and the center level. Individual respondents who returned blank surveys (n = 3) were excluded from the analyses. Additionally, responses from 2 centers were excluded due to conflicting responses from the medical and surgical directors regarding whether patients were evaluated for ELT. We performed a center-level analysis of responses to questions regarding practices related to ELT. For centers where responses were received from both the medical and surgical directors (n = 10), an a priori procedure was used to analyze these responses in which the more inclusive response was used to represent that particular center if there was a difference in the criteria or factors considered for listing. This procedure carries with it the assumption that given the scarcity of the resource being distributed, center directors would take a larger number of factors into consideration when deciding how it was distributed. Analyses were performed using R for Windows (Version 4.2.0/Vienna, Austria). Center characteristics and responses to questions on attitudes toward ELT were compared using Chi-squared tests for categorical variables and Wilcoxon rank-sum tests for continuous variables. Two-sided *P* values with an alpha of 0.05 were used and reported as per the method of Louis and Zeger.^19^

## RESULTS

### Study Population

The survey was distributed to 246 medical and surgical directors at 123 different centers. Completed responses were received from 27.2% (n = 67) of directors and 45.5% (n = 56) of centers, with responses from all 11 OPTN regions. There were no statistically significant differences in the number of LTs performed in the year preceding the survey between responding centers compared with centers from which no response was received (Table 1). Among the respondents, 47.8% (n = 32) were transplant hepatologists, whereas 52.2% (n = 35) were transplant surgeons.

**TABLE 1.** Center characteristics for survey responders compared with those of nonresponders

	Survey nonresponders	Survey responders	*P* value^*a*^
N	67	56	
Number of liver transplants performed (median [IQR])	60.00 [29.50, 98.50]	82.50 [36.75, 118.25]	0.085
OPTN region (%)			0.319
1	2 (3.0)	4 (7.1)	
2	7 (10.4)	9 (16.1)	
3	11 (16.4)	6 (10.7)	
4	4 (6.0)	9 (16.1)	
5	6 (9.0)	5 (8.9)	
6	4 (6.0)	2 (3.6)	
7	8 (11.9)	4 (7.1)	
8	4 (6.0)	4 (7.1)	
9	6 (9.0)	4 (7.1)	
10	7 (10.4)	7 (12.5)	
11	8 (11.9)	2 (3.6)	

^*a*^Continuous variables compared using Wilcoxon rank-sum test and categorical variables compared using Fisher’s exact test.

OPTN, Organ Procurement and Transplantation Network.

### Center Practices and Circumstances for Considering ELT

All centers (n = 56) indicated that they evaluate patients who have <6 mo of abstinence for LT, and 3.6% (n = 2) of centers reported enforcing any minimum abstinence time requirement for LT. With regard to candidates evaluated for ELT annually, over half (62.5%, n = 35) of centers reported evaluating >10 candidates. With regard to the annual number of ELT recipients, less than a fifth (18.2%, n = 10) of centers reported >10 recipients (Table 2). The most frequently reported circumstance under which patients were considered for ELT was having <6 mo of anticipated survival without a transplant (96.4%, n = 54), followed by an inability to participate in alcohol addiction therapy due to the acuity of illness (75.0%, n = 42). One center did not offer ELT; the reasons provided for not doing so were “a high relapse rate” and “an organizational opinion that ELT was unfair.”

**TABLE 2.** Center practices regarding ELT

Number of candidates evaluated for ELT annually (%)^*a*^	
1–5	8 (14.3)
6–10	13 (23.2)
>10	35 (62.5)
**Number of patients receiving ELT annually (%**)	
1–5	25 (44.5f)
6–10	21 (38.2)
>10	10 (18.2)
**Criteria for listing patients for ELT**	
Social support (%)	56 (100.0)
Previous alcohol-associated legal consequences (%)	53 (94.6)
Social worker evaluation (%)	52 (92.9)
Insight into previous drinking history (%)	52 (92.9)
No comorbid psychiatric disease (%)	51 (91.1)
Knowledge of previous alcoholic liver disease (%)	46 (82.1)
Evaluation by a substance use provider (%)	40 (71.4)
Written abstinence contract (%)	35 (62.5)

^*a*^Percentage of responding centers.

ELT, early liver transplant.

### Listing Criteria For ELT

All centers (100%, n = 56) considered the strength of social support as a criterion in listing candidates for ELT. More than 90% of centers considered a lack of previous alcohol-associated legal consequences, formal evaluation by transplant social workers, and insight into harmful drinking. Only 62.5% (n = 35) of centers considered the signing of a written abstinence contract as a criterion in listing candidates for ELT.

Of the responding center directors, 66.1% (n = 37) indicated that their institution had established criteria for listing candidates with severe ALD for ELT. Regarding important factors to consider when listing candidates for ELT, 57.1% (n = 32) indicated that patient survival was important, 37.5% (n = 21) indicated graft survival was important, and 55.4% (n = 31) indicated that the risk of relapse was important to consider. 7.1% (n = 4) indicated that it was unfair to offer LT to patients with severe ALD, and 25% (n = 14) indicated that ELT disadvantaged other waitlist candidates. 12.7% (n = 7) indicated that having 6 mo of pretransplant alcohol abstinence decreased the risk of posttransplant relapse, 85.5% indicated that a 5% risk of relapse was appropriate for listing candidates for ELT, 32.4% indicated that 25% was appropriate, and 3.6% indicated that a 50% risk was appropriate (Table 3). Of note, 39% of centers reported having pretransplant weight-loss criteria for listing patients with NASH, this came in the form of mandatory weight loss or signing a weight-loss contract pretransplant.

**TABLE 3.** Center attitudes regarding ELT

Survey question	Agree (%)^a^	Neutral (%)^a^	Disagree (%)^a^
**Our center has established criteria for ELT**	37 (66.1)	11 (19.6)	8 (14.3)
**The most important factor to consider when listing for ELT is:**			
** Patient survival**	32 (57.1)	16 (28.6)	8 (14.3)
** Graft survival**	21 (37.5)	26 (46.4)	9 (16.1)
** Risk of relapse**	31 (55.4)	16 (28.6)	9 (16.1)
**Offering ELT is unfair to other candidates on the waitlist**	4 (7.1)	7 (12.5)	45 (80.4)
**ELT disadvantages other candidates on the waitlist**	14 (25.0)	24 (42.9)	18 (32.1)
**6 mo of abstinence decreases the risk of relapse**	7 (12.7)	22 (40.0)	26 (47.3)
**5% risk of relapse is appropriate for listing**	48 (85.7)	5 (8.9)	3 (5.4)
**25% risk of relapse is appropriate for listing**	21 (38.2)	20 (36.4)	14 (25.5)
**50% risk of relapse is appropriate for listing**	2 (3.6)	10 (18.2)	43 (78.2)

^a^Percentage of responding centers.

ELT, early liver transplant.

### Center Director Attitudes Toward ELT

When attitudes toward ELT were compared between medical and surgical directors, a higher proportion of surgical directors indicated that their center had effective systems to monitor post-LT relapse (70% versus 51%, *P* < 0.01), and a higher proportion of medical directors indicated that ELT disadvantages other waitlist candidates (33% versus 22%, *P* = 0.03).

Open-ended comments on ELT were received from 13 survey respondents. A third of the comments (n = 4) related to the ethics of ELT. Three of the 4 comments noted the stigmatization of ALD (eg, as a result of “moral failure”) and the unfair treatment of individuals with ALD when juxtaposed with other comparable patient populations, such as hepatitis C and NASH. One of the 4 comments highlighted that the practice of ELT could be seen as irresponsible without a “robust psychosocial team for evaluation” and posttransplant support for alcohol abstinence.

Two open-ended comments raised distinct concerns about ELT. One comment mentioned that centers may disregard ELT criteria to increase throughput. Another noted that broadening medical criteria for ELT has resulted in higher relapse and graft loss, which have upset addiction counselors. The remaining comments addressed specific center practices (eg, “case-by-case basis” of ELT or use of “Dallas criteria”) and further thoughts regarding ELT (eg, ELT as “not perfect” or “uncharted territory” for the center).

## DISCUSSION

Our national study assessed transplant center practices and provider attitudes regarding ALD at different centers across the United States. We found a considerable increase in the proportion of surveyed centers performing ELT compared with studies by Hasanin et al and Bangaru et al.^11,12^ Most respondents indicated that their center evaluated >10 candidates for ELT annually and transplanted 1–5 candidates annually. Only 12.7% of the responding center directors believed 6 mo of abstinence reduced the risk of alcohol relapse post-LT. This is aligned with recent observational study data showing no association between a 6-mo abstinence period and a return to alcohol use after LT/ELT.^20–23^ Although a previous study of individuals with ALD showed a decreased risk of post-LT alcohol use with increasing duration of pretransplant abstinence, the optimal period remains unclear, and a 6-mo convention is arbitrary.^24^ The American Association for the Study of Liver Diseases, which previously endorsed a 6-mo abstinence period, now notes that a “6-month rule” is an inadequate predictor of return to drinking after LT and that there is a diminishing consensus regarding the appropriateness of this rule.^25^ But despite growing experience and familiarity with ELT and awareness of the evidence supporting this practice, respondents to our survey still indicated that ELT is controversial in their center.

The controversy around ELT arises from perceived tension between the duty to offer potentially life-saving treatment to individual patients, reflected in our study by the priority given to candidates with <6 mo of life expectancy without a transplant, and the duty to responsibly steward the limited supply of deceased donor organs for all patients, reflected in our study by the stringent criteria applied for selection of candidates for ELT. Interestingly, a larger proportion of respondents indicated that the risk of posttransplant relapse was an important factor in candidate selection compared with those that indicated graft survival was important, suggesting that relapse causing premature loss of an allograft that might have been offered to another candidate is not the only concern. Additionally, a risk of relapse between 5% and 25% appears to be most acceptable among providers as evidenced by the decrease in the proportion of respondents agreeing that these levels of relapse were acceptable for ELT. These findings, in addition to the insights from open-ended comments, may reflect concerns that relapsing to alcohol use after transplant might place added burdens on the health system or negatively affect recipients’ quality of life. Alternatively, these findings may indicate concerns that any posttransplant relapse in itself represents a misuse of the transplant and inefficient use of the organ as a scarce resource, even if it does not result in graft loss.

Although most respondents felt the 6-mo rule was a flawed predictor of posttransplant relapse, identifying candidates with a higher likelihood of sustained abstinence was clearly a priority. When evaluating candidates, considerations that weighed unfavorably included prior “wake-up calls” in the form of knowledge of ALD or legal consequences of alcohol use that did not lead to the candidate abstaining from alcohol. In contrast, insight into harmful alcohol use weighed favorably in nearly all centers. Less than half of the responding centers indicated that they utilized pretransplant weight-loss criteria in evaluating LT candidates with end-stage liver disease secondary to NASH, a disease process with a behavioral component similar to that of ALD. This discongruence in pretransplant evaluation practices merits further consideration given this similarity in the influence of behavior on outcomes in these 2 causes of end-stage liver disease.

Respondents universally indicated that social support was an important factor in decisions about listing candidates for ELT. Although social support may be considered in all transplant listing decisions, it is particularly scrutinized for patients with ALD.^12^ Poor social support in the form of having no spouse or having a subjectively poor marital relationship is a strong predictor of return to alcohol use posttransplant.^26–28^ Social support criteria have been criticized as subjective and potentially compounding challenges faced by groups disadvantaged in transplantation. The availability of social support varies based on socioeconomic status (SES) and educational attainment.^29^ A previous study showed that adults with lower levels of education and income report lower levels of support from spouses, partners, and friends.^30^ Living in adverse conditions can foment interpersonal tensions, hindering the development of relationships that can provide consistent support during difficult life experiences. Individuals of low SES have networks primarily consisting of others who are also subjected to stressful life events and are therefore less able to provide support. When these factors are considered, the more stringent use of social support as a criterion for ELT could inadvertently introduce disparities into the process. Transplant centers have an opportunity to develop programs that provide additional social support for candidates in need, augmenting the existing support systems of individual patients and ultimately improving the equity in the distribution of this valuable resource.

The strengths of this study include its inclusion of both medical and surgical directors, as well as its investigation of practices and attitudes regarding ELT, distinguishing it from a recent similar study by Lim et al that only assessed center practices by surveying medical directors. These strengths provide a more comprehensive assessment of the attitudes and practices that can guide further qualitative or mixed-methods studies to further investigate differences in perception between the 2 leaders of the clinical care team. However, our study is limited by response bias, wherein respondents are more likely to be performing ELT, and the opinions discussed are not necessarily representative of the wider community of transplant providers. The generalizability of findings is also limited by the relatively low response rate. However, although only 32% of participants responded to the survey, it is similar to response rates of previous similar studies designed to assess opinions and practices surrounding ELT.^11,12^ It should also be noted that there was no formal definition of relapse provided in this survey. This could have affected how respondents interpreted the questions regarding the risk of relapse, as different extents of a return to drinking (any alcohol use versus heavy drinking) may have varying effects on outcomes such as survival and graft failure. Additionally, due to the format in which the survey was distributed, when evaluating important factors for listing ELT candidates, respondents had the ability to affirm multiple factors as “most-important.” This may have affected how respondents interpreted this question, as a result, the study team reported these responses as factors considered important for listing ELT candidates. The exclusion of pediatric transplant programs also limits the interpretation of the survey results. ELT affects the number of organs available for pediatric transplant candidates, and the opinions of transplant providers in this field may differ from those of adult LT personnel, especially as they relate to fairness and potentially disadvantaging other waitlisted candidates. To capture additional nuances, the entire comprehensive transplant team would need to be surveyed. Our study design did not permit this, but future studies involving additional team members can provide more insight into center-level variations in attitudes toward ELT.

## CONCLUSIONS

Experience and familiarity with ELT for severe ALD continue to grow among transplant centers in the United States. Candidates are selected on a case-by-case basis, according to stringent, center-specific criteria that prioritize the life-saving purpose of ELT while seeking to reduce posttransplant relapse to harmful drinking. Continued investigation into posttransplant outcomes, especially alcohol relapse, is integral to the safe implementation of this practice and the creation of a national consensus for the distribution of this valuable resource.

## Supplementary Material
