# Supplementary material for: Transplant Center Attitudes Toward Early Liver Transplant for Alcohol-associated Liver Disease
Source: Transplant Direct. 2023 Aug 28;9(9):e1532. doi: 10.1097/TXD.0000000000001532 (PMC10465102; doi:10.1097/TXD.0000000000001532)
Supplement: Supplementary file 1 [file txd-9-e1532-s001.pdf]

SUPPLEMENT 1

Early Liver Transplantation for Alcohol-associated Liver Disease National Survey

Start of Block: Default Question Block

Q1 We invite you to participate in a very short (approximately 5-minute) survey assessing transplant center attitudes and practices regarding early liver transplantation (prior to six months alcohol abstinence) for patients with alcohol-associated live disease. Your return of this survey is implied consent. By continuing, you consent and agree to the following:

1) Your participation in this study is voluntary and will involve completing an online survey. The risks to you as a participant are minimal. These include feeling uncomfortable to provide answers according to your clinical practice. However, you may choose to skip any questions that make you uncomfortable.
2) The results of this study may be published in scientific research journals or presented at professional conferences. However, your name and identity will not be revealed, and your record will remain anonymous.
3) Participation in this study is not compensated and will not benefit you directly, but your participation may benefit others by informing transplant practices.
4) You can choose not to participate in this survey. If you decide not to participate, there will not be a penalty to you or loss of any benefits to which you are otherwise entitled. You may withdraw from this study at any time.
5) Your decision to participate in the research will not affect (favorably or unfavorably) performance evaluations, career advancement, or other employment-related decisions made by peers or supervisors. you have questions about your rights as a research participant, you can call the Johns Hopkins University Institutional Review Board at 410-502-2092 and reference [00282599]. *Thank you. We value and appreciate your time!*

○ I Agree (1)

Q2 What is your role on the transplant team?

- ○ Transplant Surgeon (1)
- ○ Transplant Hepatologist (2)
- ○ Licensed Social Worker (3)
- ○ Transplant Surgery Fellow (4)
- ○ GI/Hepatology Fellow (5)
- ○ Nurse Coordinator (6)
- ○ Physician Assistant (7)
- ○ Other (enter below) (8) ____

Q3 Does your center evaluate transplant candidates for early liver transplantation for alcohol associated liver disease (prior to 6-months of alcohol abstinence)?

- ○ Yes (1)
- ○ No (2)

Display This Question:

If Does your center evaluate transplant candidates for early liver transplantation for alcohol assoc... = Yes

Q5 Under which circumstances is early liver transplantation considered? Select all that apply:

- ▢ Patient is unlikely to survive 6-months without liver transplant (1)

☐ Patient us unable to participate in alcohol use disorder therapy due to severity of illness (2)

☐ Patient has a living donor (3)

☐ Other (enter below) (4)
____

Display This Question:

If Does your center evaluate transplant candidates for early liver transplantation for alcohol assoc... = Yes

Q6 Is there a minimum length of abstinence time required?

○ Yes (1)

○ No (2)

Display This Question:

If Is there a minimum length of abstinence time required? = Yes

Q7 How many months of abstinence are required at your center?

____

Display This Question:

If Does your center evaluate transplant candidates for early liver transplantation for alcohol assoc... = Yes

Q8 Approximately how many patients are evaluated for early liver transplantation per year?

- ○ 1-5 (1)
- ○ 6-10 (2)
- ○ >10 (3)

Display This Question:

If Does your center evaluate transplant candidates for early liver transplantation for alcohol assoc... = Yes

Q9 Approximately how many patients undergo early liver transplantation per year?

- ○ 1-5 (1)
- ○ 6-10 (2)
- ○ >10 (3)

Display This Question:

If Does your center evaluate transplant candidates for early liver transplantation for alcohol assoc... = Yes

Q10 What criteria does your center use to select candidates for early liver transplantation? Select all that apply:

- ▢ Strength of social support (1)
- ▢ Knowledge of previous alcohol-associated liver disease (2)
- ▢ Previous failed enrollments in alcohol rehabilitation programs (3)
- ▢ Written abstinence contract (4)
- ▢ Lack of severe comorbid psychiatric disease (5)
- ▢ Formal evaluation by substance use disorder provider (6)
- ▢ Formal evaluation by transplant social worker (7)

- [ ] Candidate level of insight into harmful drinking history (8)
- [ ] Age younger than xx (enter below) (9) ____
- [ ] Previous alcohol-associated legal consequences (10)
- [ ] Other (enter below) (11) ____

Display This Question:

If Does your center evaluate transplant candidates for early liver transplantation for alcohol assoc... = No

Q11 What are the main reasons your center does not consider early liver transplantation for alcohol-associated liver disease? Select all that apply:

- [ ] Do not receive referrals for patients with less than 6 months abstinence (1)
- [ ] Risk of decreased donation rates due to public opinion (2)
- [ ] Organizational opinion that it is unfair to allocate a limited resource to these patients (3)
- [ ] Risk of high alcohol relapse rates (4)
- [ ] Risk of poor post-transplant patient or graft survival (5)
- [ ] Lack of infrastructure to treat alcohol use disorder after transplantation (6)
- [ ] Other (enter below) (7) ____

Display This Question:

If Does your center evaluate transplant candidates for early liver transplantation for alcohol assoc... = No

Q12 Does your center refer patients with less than 6 months abstinence to a different center if they are unlikely to survive 6 months without transplant?

- ○ Yes (1)
- ○ No (2)

Q14 Does your center require pre-transplantation weight loss or behavioral contract requirements for candidates listed for transplantation due to non-alcoholic fatty liver disease?

- ○ Yes (1)
- ○ No (2)

Display This Question:

If Does your center require pre-transplantation weight loss or behavioral contract requirements for... = Yes

Q15 What requirements does your center have for these patients? Select all that apply:

- ☐ Pre-transplant mandatory weight loss (1)
- ☐ Behavioral contract (2)
- ☐ Other (enter below) (3) ____

Display This Question:

If Does your center evaluate transplant candidates for early liver transplantation for alcohol assoc... = Yes

Q17 Use the scale below to indicate the centers agreement or disagreement with the following statement:

	Disagree (1)	Neither Agree Nor Disagree (2)	Agree (3)
Our center has clear established criteria for deciding to list a patient with alcohol-associated liver disease for early liver transplantation. (1)	○	○	○

Q16 Use the scale below to indicate the centers agreement or disagreement with the following statement:
In evaluating a patient with Alcohol-associated liver disease for transplant...

	Disagree (1)	Neither Agree Nor Disagree (2)	Agree (3)
Patient survival is the most important factor considered for listing. (2)	○	○	○
Graft survival is the most important factor considered for listing. (3)	○	○	○
Risk of alcohol-relapse is the most important factor considered for listing. (4)	○	○	○

Q21 Use the scale below to indicate the centers agreement or disagreement with the following statements:

	Disagree (1)	Neither Agree Nor Disagree (2)	Agree (3)
Our center has an effective evaluation system to assess patients' risk of post-LT alcohol relapse. (1)	○	○	○
It is unfair to offer liver transplant to patients with Alcohol-associated liver disease prior to demonstrating 6-months abstinence. (5)	○	○	○
Early liver transplantation for patients with Alcohol-associated liver disease disadvantages other patients on the wait list. (6)	○	○	○
Our center has clear established criteria for deciding to list a patient with alcohol-associated liver disease for LT. (7)	○	○	○
Having at least 6-months of pre-transplantation alcohol abstinence decreases the risk of	○	○	○

post-LT alcohol
relapse. (8)

Q20 Use the scale below to indicate the centers agreement or disagreement with the following statements:
It is appropriate to list a patient with alcohol-associated liver disease for LT if they have...

	Disagree (1)	Neither Agree Nor Disagree (2)	Agree (3)
A 5% risk of alcohol relapse (1)	○	○	○
A 25% risk of alcohol relapse (4)	○	○	○
A 50% risk of alcohol relapse (5)	○	○	○

Q18 Does your center offer post-LT alcohol use disorder treatment services as part of the standard post-LT follow up for alcohol associated liver disease patients?

○ Yes (1)

○ No (2)

Q19 Drag and drop to choose up to three words/phrases from the list below that best represent your centers view of early liver transplantation (prior to 6-months abstinence) for alcohol-associated liver disease:

Represents my centers view of early liver transplantation

_______ Risky (1)

_______ Life-Saving (2)

_______ Irresponsible (3)

_______ Standard-of-care (4)

_______ Waste of a limited resource (5)

_______ Controversial (6)

_______ Unnecessary (7)

_______ Common Practice (8)

_______ Underutilized (9)

_______ Subjective (10)

_______ Unfair (11)

_______ Accepted (12)

_______ Safe (15)

_______ Fair (16)

_______ Other (17)

Q20 If you have any additional comments regarding early liver transplantation for alcohol-associated liver disease at your center, please provide them below:

____

End of Block: Default Question Block
